# The Non-Invasive Transcranial Doppler for Hemodynamic Monitoring

**DOI:** 10.33549/physiolres.935413

**Published:** 2025-06-01

**Authors:** Peter SCHEER, Jana HLOŽKOVÁ, Eliška BRHELOVÁ, Ahmet Davut AKSU, Simona GOLIÁŠOVÁ, Jana DOLEŽALOVÁ, Lada TLUČHOŘOVÁ, Robert MIKULÍK

**Affiliations:** 1 International Clinical Research Center, St. Anne’s University Hospital, Brno, Czech Republic; 2 Faculty of Pharmacy, Masaryk University, Brno, Czech Republic

**Keywords:** Non-invasive monitoring, Brain flow velocity, Anesthesia, Animal model

## Abstract

The successful development and testing of new thrombolytics in animal models requires monitoring of hemodynamic changes in cerebral circulation before and after stroke. The purpose of the present study was to document that percutaneous transcranial Doppler (TCD) monitoring is able to differentiate two hemodynamic situations induced with two anesthetic protocols. Twelve adult rats divided into two groups underwent general anesthesia (60 min) using combination: 1) ketamine-xylazine-diazepam (KXD); and 2) ketamine-xylazine-urethane-alpha-chloralose (URACH). The TCD was performed with the skin and skull intact. The heart rate, peak systolic velocity, pulsatility index, and resistance index were recorded in a branch of the posterior cerebral artery. Flow detection and measurement was possible in all rat brains bilaterally. The mean heart rate was lower in the KXD 243±4 (range: 238 to 249) than in the URACH group 265±12 (range: 250 to 279), the difference between means: 22; 95 % CI [8 to 34], *p*=0.005) only for the first 20 min of monitoring. Peak systolic velocity was lower in the KXD 73.4±3.3 mm/s (range 70.3 to 76.5) vs. URACH group 93.7±4.0 mm/s (range: 90.0 to 97.4) during the entire observation period (difference between means: 20; 95 % CI [16 to 25], *p*<0.001). Same difference was observed for pulsatility and resistance indexes. TCD was able to differentiate hemodynamic changes in the rat brains, making the TCD suitable for monitoring of hemodynamic changes and explores, e.g. how such changes contribute to hemorrhagic transformation after thrombolysis. Also, TCD holds promise as a tool for monitoring of recanalization induced by thrombolytics.

## Introduction

Intravenous thrombolysis is the first-choice therapy for patients with acute ischemic stroke [1]. Despite the relative ease of use and benefits of thrombolysis, there is potential to improve the efficacy and safety of thrombolysis: recanalization is achieved in 4–44 % [2] of stroke patients and 3–40 % were identified with the hemorrhagic transformation [3,4]. Collateral blood flow support is one way to increase the brain’s post-ischemic surveillance [5]. However, the hemodynamic changes can account for hemorrhagic transformation [4], thus, drug efficacy in brain circulation, including the monitoring of the hemodynamic changes of the blood flow, would be needed.

Most monitoring techniques used in stroke animal models (i.e., laser Doppler flowmetry, laser speckle contrast imaging, optical coherence tomography, etc.) can detect vessel embolic occlusion and measure blood flow [6–8]. However, they require invasive surgical (i.e., thinning or removal of part of the skull), or terminal procedures or young animals with thin skull bones [9], limiting their value in the stroke setting [10]. Almost identical to clinical usage, magnetic resonance perfusion imaging [11] would be ideal choice. However, the costs could limit the availability. Transcranial Doppler (TCD) has been used in clinical medicine for more than five decades, including the management of stroke patients [12]. The use of TCD in preclinical research is underestimated, although its availability, transferability, and possibility of repeated non-invasive bedside testing [13].

In stroke animal models, general anesthesia is required and its influence on cerebral blood flow should be evaluated [6–8,13]. However, the study of ideal anesthetic combinations for stroke research with minimum effect on hemodynamics and/or function of other organ systems is missing. Therefore, the aim of the study was to assess the ultrasound transcranial Doppler system’s ability to monitor the difference of blood flow in distal branches of cerebral arteries of adult rats caused by two anesthetic combinations. Velocity parameters were measured to discern the difference in blood flow changes. All measurements were without causing harm to the skin and skull.

## Material and Methods

### Consent for animals used in experiment

All procedures and animal experiments were performed in full compliance with the ARRIVE and the European Community Council Directive (2010/63/EU) for Protection of Vertebrate Animals Used for Experimental and other Scientific Purposes guidelines. The project was approved by the Masaryk University Brno Institutional Committee for the Protection of Experimental Animals and by the Committee for the Protection of Experimental Animals of the Ministry of Education Youth and Sports in the Czech Republic (MEYS CZ) with protocol number MSMT-10589/2022-4.

### Animals

Male outbreed Wistar rats (n=12; bodyweight 290–522 g) were included into the study. Before the experiment, rats were individually kept under standard conditions with a natural light mode and access to food and water *ad libitum*. The housing of animals and morning surgical procedures were performed at the Pharmaceutical Faculty, Masaryk University, Brno.

### Experimental design and protocol

For the study purposes, commonly used combination ketamine and xylazine were chosen for its safe, effective anesthesia and ability to prolong the anesthetic protocol [14,15]. These were combined with diazepam, which effect on hemodynamic changes in brain was not well studied or urethane with alpha-chloralose, well studied anesthetics in neurology [16–18], but not in combination with ketamine and xylazine.

The rats were randomly assigned (simple randomization by drawing lots by the animal caretaker) to two anesthetic protocols with: a) KXD – a combination of **K**etamine-**X**ylazine-**D**iazepam, and b) URACH – a combination of ketamine-xylazine with **UR**ethane and **A**lpha-**CH**loralose.

KXD protocol: rats were under general anesthesia induced by the initial inhalation of 2.5 % isoflurane in the carrier gas (filtered room air). During the isoflurane induction, xylazine and ketamine were injected intramuscularly (5 mg/kg and 35 mg/kg of body weight, respectively), and diazepam (2 mg/kg of body weight) was applied into the peritoneal space.

URACH protocol: induction of general anesthesia using isoflurane, ketamine, and xylazine were proceed as mentioned above. After ketamine and xylazine application, a mixture of urethane (80 mg/ml) and alpha-chloralose (6 mg/ml) in a dose of 0.8 ml per 100 g of body weight was applied into the peritoneal space.

In both groups during first 15 min of protocol, the intravenous (IV) cannula (Vasovet 21 G, B. Braun) was inserted into tail vein in.

### Transcranial Doppler setting and measurement

**Fig. 1:**
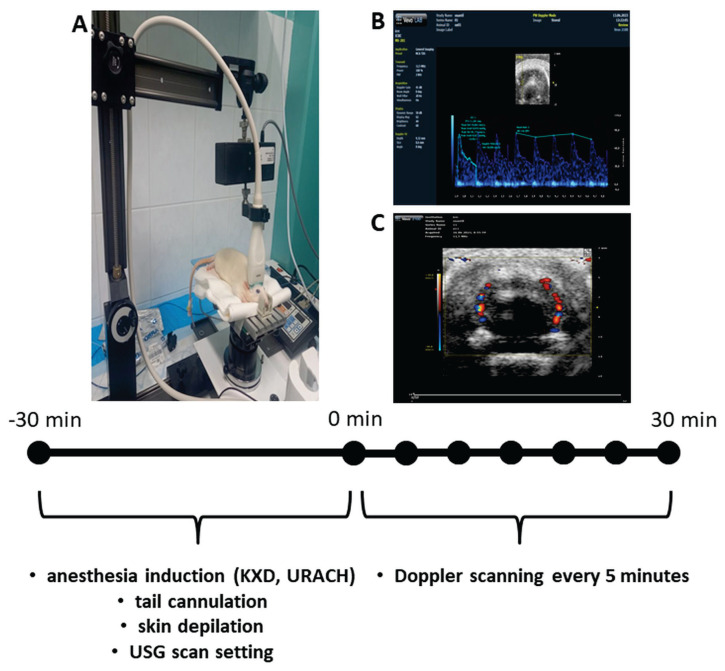
Timeline of 60 min experimental procedure used for the transcranial Doppler monitoring. During the first 30 min, the rats were anesthetized and fixed for monitoring (**A**). At the time 0 min started the flow velocity monitoring (**B**) in distal branches of supracollicular arterial network (**C**).

The fur above the parietal area of the skull was clipped and the remaining fur was removed using depilatory cream. After the insertion of the IV cannula into tail vein, the anesthetized rats were transferred on a heated examination table. To prevent aspiration of saliva or stomach content during the anesthesia, the table was tilted by five degrees with the head upside down in the longitudinal axis. The head was fixed in a retarder to prevent its movement during the examination (Fig. 1). Tempered ultrasound conduction gel was applied to improve the conductivity. Using a VEVO 2100 (VisualSonic) device with an MS201 probe, the rats were examined. The probe was placed above the skull and the cross-sectional scan was performed by moving the probe from back to frontal till identification of posterior cerebral arteries (PCA). Different mode of device was set up for color flow and pulse wave measurement in posterior cerebral artery branches.

Setup for *color flow Doppler* mode was frequency 12.5 MHz, power 100 %, pulse repetition frequency (PRF) 1 kHz, Gate 4, Doppler gain 23 dB, 2D gain 39 dB, frame rate 9, depth 15 mm, width 25 mm, sensitivity 5, line density full, persistence off, respiratory gate off, extended buffer off, wall filter high, and priority 80 %.

Setup for *pulse wave measurement* was frequency 12.5 MHz, power 100 %, PRF 2 kHz, Doppler gain 41 dB, wall filter 20 Hz, dynamic range 50 dB, depth 7.65 mm, Doppler – size 0.6, and angle 0 degree.

First, the coronal section at the level of internal capsule in 2D mode was visualized. Using the color flow Doppler mode, the blood stream in supracollicular arterial network (which are PCA branches) identified [19]. Pulse flow Doppler mode was used for flow velocity recording.

In a place with maximum flow signal, first, pulse flow was recorded in the right and then in the left supracollicular arterial network.

The ultrasound record was saved at time 0 min that was 30^th^ min of anesthetic protocol. Then every 5 min, the blood flow velocity was recorded from time 0 to time 30 min (time-line is in Fig. 1). The ultrasonic records were randomized to the group (performed by JH) and as well blinded for evaluators (LT, SG) for following analyses.

### Flow Velocity records analysis

Doppler curves were analyzed in VevoLAB 1.7.0 software (VisualSonic). Using the protocol for Doppler record analysis, the variables of spectral waveform as the peak systolic velocity (PSV; mm/s), end-diastolic velocity (EDV; mm/s), and mean flow velocity (MFV; mm/s) were measured. The pulsatility index (PI; Gosling index) and resistance index (RI, Pourcelot index) were calculated using the velocity parameters (PSV, EDV, and MFV) [20]. The heart rate (HR) was measured from 5 heart cycle (=velocity wave). All analyses were performed by 2 persons (SG, LT) blinded to each other’s readings and to animal group allocation.

### Statistics

All data of HR, PSV, PI, and RI were expressed as the mean and standard deviation (SD) for each time point within each tested group. The basic parameters of descriptive statistics were calculated. Unpaired parametric *t*-test was used to assess the statistical difference in velocity parameters between the tested groups. Statistical significance was set at *p*<0.05. The analyses were done using GraphPad Prism 8.4.2 (GraphPad Software, San Diego, California, USA), performed by EB, ADA.

Agreement on measurements of velocity parameters by two evaluators was examined by intra-class correlation coefficient (ICC). ICC estimates and 95 % confidence interval were calculated using SPSS statistical package version 23 (SPSS Inc., Chicago, IL) based on a mean-rating (k=2), absolute agreement, 2-way mixed-effects model.

All animals were included in analyses with no animals excluded for any reason.

## Results

The obtained ICC value for mean PSV in KXD and URACH group was 0.70 (95 % CI 0.03 to 0.94) and 0.74 (95 % CI 0.03 to 0.94), respectively. The level of reliability between two evaluators is good to very good [21].

**Fig. 2:**
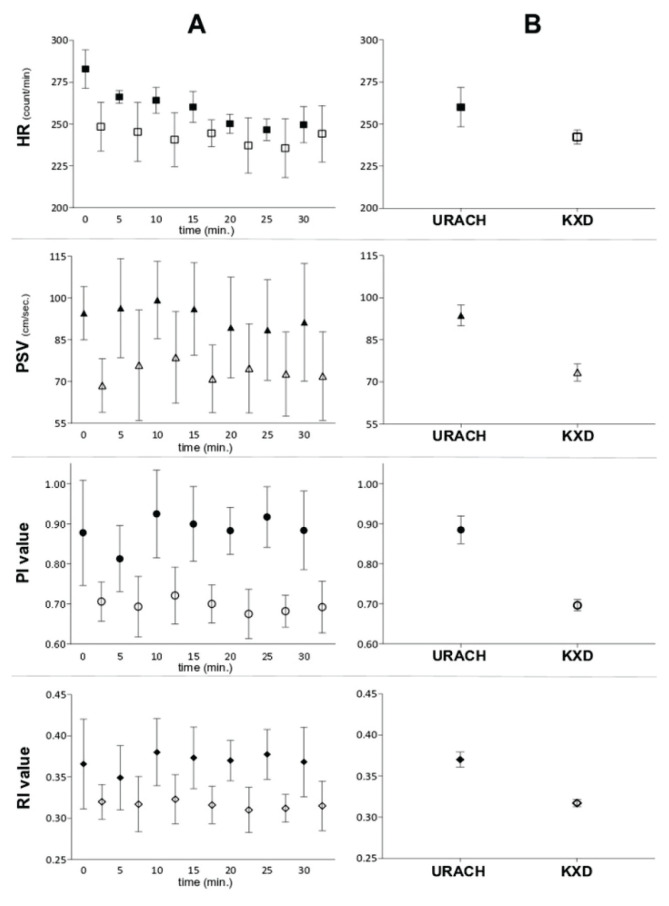
Effect of anesthetics on variables of spectral waveform. A column – the graphical presentation of mean values and 95 % confidence intervals at each time point of experimental protocol (X axis) for KXD (empty geometric shapes) and URACH (full geometric shapes) group; B column – the graphical presentation of mean values and 95 % confidence intervals of analyzed parameters for each entire anesthetic protocol. KXD=ketamine-xylazine-diazepam; URACH=ketamine-xylazine-urethane-alpha-chloralose; HR=heart rate; PSV=peak systolic velocity; PI=pulsatility index; RI=resistance index.

During first 20 min of the anesthetic protocol, the mean heart rate (HR; beats/min.) was significantly lower in the KXD (mean: 243±4; range: 238 to 249) than in the URACH group (mean: 265±12; range: 250 to 279), absolute difference between the means: 22; 95 % CI [8 to 34], *p*=0.005). From the twentieth minute of the protocol, the HR in the URACH group approached the proximity of the KXD group (Fig. 2), and at 30^th^ min, the mean HR value was in the KXD group 244±24 (range 227 to 261) and in the URACH group 250±17 (range: 239 to 260) (absolute difference between the means: 5; 95 % CI [−13 to 23], *p*=0.540).

Also mean peak of systolic velocity (PSV; mm/s) was significantly lower in the KXD (73.4±3.3; range 70.3 to 76.5) than in the URACH group (93.7±4.0; range: 90.0 to 97.4) during the entire observation period (absolute difference between the means: 20; 95 % CI [16 to 25], *p*<0.001). The highest average peak of systolic velocity in both groups was detected at 10 min of the protocol (= 40 min from anesthesia induction).

The values of end diastolic velocity (EDV; mm/s) did not differ between the KXD (38.7±1.9; range 37.0 to 40.5) vs. the URACH group (41.9±2.5; range: 39.6 to 44.2) during the entire observation period (absolute difference between the means: 3.2; 95 % CI [0.6 to 5.8], *p*=0.018).

The dynamic change of pulsatility (PI) and resistance index (RI) showed the same change as PSV during the monitored period. The mean PI in the KXD vs. URACH group was 0.70±0.02 vs. 0.88±0.04 (absolute difference between the means: 0.19; 95 % CI [0.16 to 0.22], *p*<0.001) and RI in the KXD vs. URACH group was 0.32±0.01 vs. 0.37±0.01 (absolute difference between the means: 0.05; 95 % CI [0.04 to 0.06], *p*<0.001). The highest average peak of both indices was also at 10 min of the analyzed period (Fig. 2).

## Discussion

To validate the TCD capability of hemodynamic monitoring, we conducted comparative analyses on the hemodynamic changes in brain arteries induced by two combinations of anesthetics. A specialized high-resolution ultrasound tomograph, the VEVO2100 designed for small animals, achieved a 100 % success rate in registration and measurements of hemodynamic parameters in the second-order arterial branches of the circle of Willis. The TCD system allowed the monitoring also in relatively large adult rats, which are often overlooked in stroke research due to their body size being incompatible with commonly used devices such as micro-CT, MRI, and ultrasound [22].

The present study showcases a novel application of TCD that eliminates the need for a contrast medium, enabling repetitive monitoring of dynamic changes even in smaller and distal branches of the main arteries of the circle of Willis, including the anterior, middle, and posterior cerebral arteries. This is because previous studies have primarily employed TCD only for measuring hemodynamic changes after intracerebral hemorrhage, for ischemic stroke or hypoxia monitoring, or for assessing the effects of vasoactive substances during migraine [23–26]. Existing approaches also often focus on thin areas of the skull, such as the temporal or occipital regions [26,27]. Only few studies included a transcranial non-invasive approach, such as a contrast-enhanced ultrasound (CEU) [10] or functional ultrasound (fUS) [28] monitoring, but with the necessity to involve additional procedures like microbubble infusion. Our study therefore documents expanded capability of TCD. This positions TCD as a comprehensive tool for non-invasive assessment across various regions of the skull, implacable for evaluation of new thrombolytics in stroke research and also in future studies where photoacoustic imaging for cerebral perfusion is needed [27].

Our analyses revealed that TCD differentiated hemodynamic changes induced by two anesthetic protocols. During the initial 20 min, HR was 8 % higher with URACH than KXD. PSV, PI, and RI were consistently 22 %, 23 %, and 16 % higher, respectively, with URACH throughout monitoring. Comparative studies on PSV vary, from 5.1 mm/s in small brain arteries in adult rats (isoflurane anesthesia) [23] and 16 cm/s in rat pups (isoflurane anesthesia) [29] to 64 cm/s in carotids of adult rats (ketamine-xylazine anesthesia) [24], which was almost 7 times higher than the highest mean PVS in present study. Only one study, reports PVS in posterior arteries 5.12–5.25 cm/s [30], which close to our results. The reporting of PI and RI indices in studies is scarce [29,31,32], but the recent study on adult rats found PI 0.09–0.33, depending on the diameter of brain artery [23]. These data are two times lower than in present study. None of the animals in the present study exceeded the values described in human subjects [20], and we assume that our velocity measurements were correct and the difference in RI and PI indices between the KXD and URACH combination were due to the vasodilatation effect of xylazine [33] in the KXD group (see below). Variability in published data may be due to factors like animal weight, anesthesia, or monitored arteries. Further research with standardized methods is needed.

The combination of anesthetics caused the difference in the velocity parameters and heart rate in the KXD and URACH protocols. Ketamine is known to increase the arterial blood pressure and HR [34], what wasn’t observed in the present study. In KXD combination, diazepam reduced the effect of ketamine on the cardiovascular system, presented here as the decrease of HR [35]. The parameters in KXD combi-nation were mainly influenced by vasodilatative effect of xylazine [15,36–37], and in combination with ketamine causes drop of the blood flow in brain compartments [38], as observed in lower PSV, RI and PI values in the KXD group. The vasodilative effect could allow the collateral circulation to open which was proven to influence recanalization during thrombolysis *in vitro* positively [39] and needs to be also evaluated in *in vitro* stroke models. As shown previously, the KXD combination provides stable anesthesia suitable for safety experiments [15,37]. In URACH combination, due to the alpha-chloralose application, the baroreflexes are preserved and arterial pressure drop is compensated with tachycardia in first 30–40 min of anesthesia (first 10 min of present monitoring window). Then the blood pressure increases gradually to normal levels [33,40] what is accompanied by a decrease in the HR [40]. The drop in HR was detected during the last 20 min of monitoring (Fig. 2). Urethane (ethyl carbamate) has similar effects to α-chloralose, but the HR remains unaffected [41,42]. Also URACH combination causes long-term anesthesia requiring prolonged monitoring [43], making it suitable for experiments testing drug’s efficacy. Due to the urethane carcinogenicity requires euthanasia post-experiment [33,44]. Moreover, the higher values of velocity parameters indicate increased blood pressure in brain arteries that could be a predictive risk factor of hemorrhagic transformation during thrombolysis in ischemic stroke models [45].

## Limitations

While the study offers valuable insights, it comes with certain limitations. These include the requirement for general anesthesia and mechanical fixation during TCD monitoring. Also, it still needs to be tested if TCD can diagnose the presence of occlusion and reperfusion in order TCD can be used to monitoring of recanalization induced by thrombolytics. And the last, no measurements of respiratory gases could limit the results interpretation. Cerebral vessels react to changes of partial pressure of CO_2_, and dilation of resistance arterioles increases the blood flow velocity [46]. These changes can potentially decrease the volume of hypoperfused tissue during an acute stroke [47].

## Conclusions

Transcranial Doppler is useful for monitoring hemodynamic changes in the rat brain in real time, making it applicable for monitoring the efficacy of thrombolytic drugs in preclinical research. The simplicity and clinical similarity of the present study represent an important step forward in achieving translational success in stroke research.
